# Genome Analysis Identifies a Novel Type III Secretion System (T3SS) Category in *Vibrio* Species

**DOI:** 10.3390/microorganisms11020290

**Published:** 2023-01-22

**Authors:** Douaa Zakaria, Shigeaki Matsuda, Tetsuya Iida, Tetsuya Hayashi, Masanori Arita

**Affiliations:** 1 Department of Genetics, SOKENDAI University, Mishima 411-8540, Japan; 2 Research Institute for Microbial Diseases, Osaka University, Suita 565-0871, Japan; 3 Department of Basic Medicine, Faculty of Medical Sciences, Kyushu University, Fukuoka 812-8582, Japan; 4 RIKEN Center for Sustainable Resource Science, Yokohama 230-0045, Japan

**Keywords:** comparative genomics, pathogen, T3SS, *Vibrio*

## Abstract

The nanomachine referred to as the type III secretion system (T3SS) is used by many Gram-negative pathogens or symbionts to inject their effector proteins into host cells to promote their infections or symbioses. Among the genera possessing T3SS is *Vibrio*, which consists of diverse species of Gammaproteobacteria including human pathogenic species and inhabits aquatic environments. We describe the genetic overview of the T3SS gene clusters in *Vibrio* through a phylogenetic analysis from 48 bacterial strains and a gene order analysis of the two previously known categories in *Vibrio* (T3SS1 and T3SS2). Through this analysis we identified a new T3SS category (named T3SS3) that shares similar core and related proteins (effectors, translocons, and chaperones) with the Ssa-Esc family of T3SSs in *Salmonella*, *Shewanella*, and *Sodalis*. The high similarity between T3SS3 and the Ssa-Esc family suggests a possibility of genetic exchange among marine bacteria with similar habitats.

## 1. Introduction

*Vibrio* is a genus of Gram-negative, facultative anaerobic, fermentative Gammaproteo-bacteria with mostly two chromosomes, living in marine and freshwater environments [1]. This genus comprises over 100 species, some of which are pathogenic and infect a variety of hosts, ranging from mammals to non-mammalian aquatic animals such as fish, mollusks, and corals. Among around a dozen of human pathogenic *Vibrio* species, the most famous is *Vibrio cholerae*, which causes widespread cholera. Several million people worldwide suffer from this illness every year, especially in developing countries. Non-cholera *Vibrio* illness, called vibriosis, is another public health concern. The Centers for Disease Control and Prevention of the United States estimates that vibriosis causes 80,000 illnesses each year within the United States alone. One of the major species causing vibriosis is *Vibrio parahaemolyticus*, which is the leading cause of seafood-borne gastroenteritis worldwide.

Type III Secretion System (T3SS) is a machinery in Gram-negative pathogens and symbionts, including *Vibrio* species, with a molecular syringe used as a protein delivery device called injectisome [2]. Using the syringe, the bacterium pierces proteins called “translocon” to form a pore in a host cell membrane. Then, proteins called “effector” are injected into the host cell and manipulate host cellular processes, such as innate immune responses, cargo trafficking, and cytoskeleton remodeling, in favor of bacterial survival and reproduction [3].

**Figure 1:**
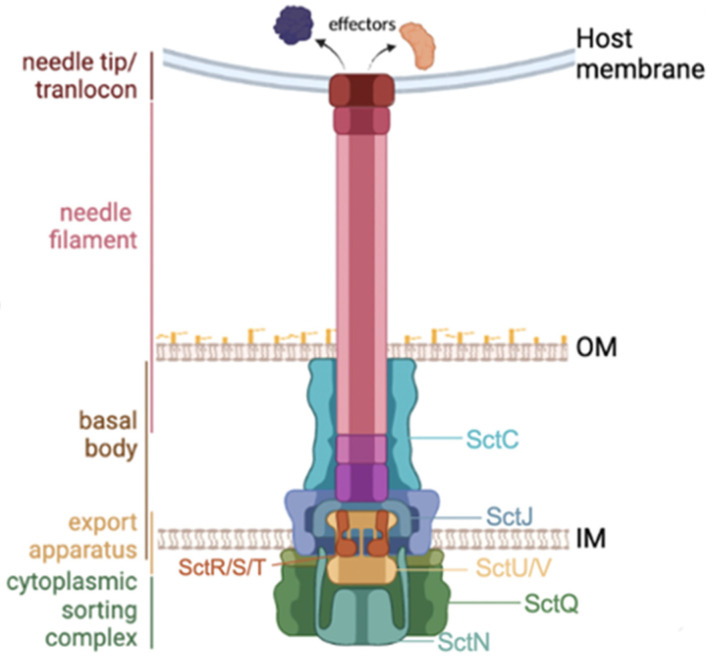
An overview of T3SS core proteins and the bacterial membranes.

The injectisome is a macromolecule composed of more than 20 proteins [4,5] and consists of four components (Figure 1): (1) an extracellular part comprising a needle and tip/translocon complex, (2) a basal body of the syringe, (3) an export apparatus formed by inner membrane proteins, and (4) a cytoplasmic complex. Among these, the basal body and export apparatus are well conserved across a wide range of Gram-negative bacteria and homologous to bacterial flagellar components [6]. To refer to these injectisome proteins, we use the unified nomenclature of Sct for “Secretion and cellular translocation” [2]. Among around 20 proteins, SctN is an ATPase that works as the secretion pump and is usually most conserved. SctQ (cytoplasmic ring), SctR-S-T-U-V (export apparatus), SctJ (inner membrane ring) and SctC (outer membrane ring) are also well conserved proteins.

Based on the sequence similarity of these proteins, T3SS is classified into nine families across several bacterial phyla [6,7]: Ysc, Inv/Mxi-Spa, Ssa-Esc, Hrp/Hrc1, Hrp/Hrc2, Rhizobiales, Chlamydiales, Myxococcales, and Desulfovibrionales (Table 1). These family names originate in species names or secreted protein names required for infection.

The first family Ysc comes from secreted *Yersinia* outer-membrane proteins. In *Yersinia*, T3SS is often encoded in a plasmid, and similar T3SSs in the Ysc family have been observed in two bacterial phyla, Alphaproteobacteria (e.g., *Bordetella*) and Gammaproteobacteria (*Yersinia*, *Pseudomonas*, *Vibrio* and others). Previously, T3SS of *Desulfovibrio vulgaris* was classified in this Ysc family but now it has become independent [8]. Upon infection, *Yersinia pseudotuberculosis* and *Yersinia enterocolitica* are known to cross the epithelium lining and stay extracellular in the mesenteric lymph nodes. T3SS effector proteins are used to avoid phagocytosis [9]. The second family, Inv/Mxi-Spa, comes from Inv-Spa and Mxi-Spa secreted protein complexes of *Salmonella enterica* and *Shigella flexneri*, respectively. This family is found in Betaproteobacteria (e.g. *Burkholderia*) and Gammaproteobacteria (Enterobacteriaceae). A well-known T3SS is encoded in *Salmonella* pathogenicity island 1 (SPI-1) of its chromosome and is associated with the invasion to host epithelial cells. The T3SS in *Shigella* is encoded in a large virulence plasmid of 230 kb. The third family, Ssa-Esc, indicates T3SSs in the SPI-2 region of *Salmonella*, in the locus of enterocyte effacement (LEE) regions of enteropathogenic/enterohemorrhagic *Escherichia coli* (EPEC/EHEC). In *Salmonella*, this T3SS is associated with later stages of infection to survive inside host cells and its mechanism is well investigated. After cell invasion, *Salmonella* forms a SCV (*Salmonella*-containing vacuole) and starts replication inside this vacuole by modulating the host cell using T3SS effectors [9]. On the other hand, EPEC/EHEC uses a single T3SS for infection and remains extracellular as *Yersinia* does [9].

The fourth and fifth families, Hrp/Hrc1 and Hrp/Hrc2, are found in plant pathogens such as *Pseudomonas syringae* and *Burkholderia pseudomallei*. The family names come from gene function as “Hypersensitive response and pathogenicity/conserved”. T3SSs in these families possess a longer injectisome for penetrating thick plant cell walls. Interestingly, some clinically isolated *Vibrio* species, including *V. cholerae* and *V. parahaemolyticus*, possess the second T3SS in this Hrp/Hrc1 family. Their length of injectisome has not been well documented.

The remaining four T3SS families (Rhizobiales, Chlamydiales, Myxococcales, and Desulfovibrionales) are order names identified through phylogenetic analyses. Among them, *Rhizobium* and *Myxococcus* are Alpha- and Deltaproteobacteria, respectively, while *Chlamydia* and *Desulfovibrio* belong to different phyla, i.e., Chlamydiota and Thermodesulfobacteria, respectively. They are outside of Pseudomonadota (formerly Proteobacteria).

The presence of T3SS in *Vibrio* was first revealed in *V. parahaemolyticus* by whole genome sequencing [10] and later its distribution in some other *Vibrio* species was also confirmed. Most *Vibrio* species possess only one T3SS gene cluster, but *V. parahaemolyticus* as well as some others possess two [10,11]. Within *V. parahaemolyticus*, the ubiquitous T3SS, referred to as T3SS1, is most similar to the *Yersinia* Ysc family and is encoded in its major chromosome. The other one, called T3SS2, is similar to the Hrp/Hrc1 family. The latter is non-essential and is encoded in a pathogenicity island called VpaI-7 in *V. parahaemolyticus*. The island is 81 kb in length and on the auxiliary chromosome, and is characterized by the presence of multiple transposase genes in contrast to the integrase genes found in the remaining 6 pathogenicity islands (VpaI-1~6) [12]. Both T3SSs of *V. parahaemolyticus* are associated with pathogenicity: T3SS1 is responsible for the cytotoxicity in cultured human cells, while T3SS2 is known to induce enterotoxicity in animal infection models and cytotoxic activity in intestinal cell lines [13,14]. So far, all *Vibrio* T3SSs have been phylogenetically classified into the above two types: one is similar to T3SS1 in the Ysc family and the other is similar to T3SS2 in the Hrp/Hrc1 family [7].

Here, we report the third type of *Vibrio* T3SS, named T3SS3, which belongs to the Ssa-Esc family and is most similar to the T3SSs in *Salmonella*, *Sodalis* and *Shewanella*. Among the T3SSs in these bacteria, the most well studied is the one located in the *Salmonella* SPI-2 pathogenicity island. The SPI-2 T3SS translocates its effectors across the membrane of SCVs after internalization, and they link the vacuole to the Golgi network of the host cell [15]. The effectors used in this process were also found in strains with T3SS3 investigated in this study.

## 2. Materials and Methods

### 2.1. Conservation Analysis

The initial dataset of T3SS core proteins was obtained from the work of Hu et al. [16]. All protein sequences were scanned for common domains using Pfam [17] and those without the common domain or those on plasmids were removed manually. To obtain T3SS protein sequences in *Vibrio* species comprehensively, the obtained T3SS core sequences were searched against *Vibrio* genomes in GenBank/ENA/DDBJ and sequences with >80% identity were selected. At this stage the sequence coverage was not considered. Then, the sequences were aligned using MAFFT [18], trimmed using trimAl [19]. The conservation rate of aligned sequences was calculated using MstatX [20].

### 2.2. Phylogenetic Analysis

Sequences of the most conserved core proteins (SctN, SctS, SctU and SctV) were aligned using MAFFT and concatenated using catfasta2phyml [21]. A phylogenetic tree of the concatenated core sequences was created using RAxML with a bootstrap value of 1000 [22]. The result was visualized using iTOL [23]. Branches of less than 70 bootstrap scores were deleted.

### 2.3. Prediction of Putative Type III Secretion Effectors (T3SEs)

Protein sequences in the twelve T3SS3 clusters were binned into homologous groups using SonicParanoid [24]. The sequences of each homologous group were aligned using MAFFT and their HMMER profile was generated. Experimentally validated T3SEs were collected from previous studies [7,15] and from the Virulence Factor Database (VFDB: http://www.mgc.ac.cn/VFs/main.htm accessed on 17 January 2023) online [25]. Protein sequences of *Aliivibrio fischeri* ES114 and *Escherichia coli* K-12 were used as the negative control set as they lack functional T3SS but possess flagella. Then, the HMMER profiles were scanned against the validated T3SEs and the negative control. Homologous groups that had no positive hit against the T3SEs dataset (E-value > 1 × 10^−3^) were dismissed from further analysis. Protein sequences in the homologous groups, whose HMMER profiles had at least one positive hit in the T3SEs dataset, were again searched in the T3SEs dataset using BLASTp. The Python script for HMMER and BLASTp evaluation was adopted from a previous study [26].

Homologous sequences that had no BLASTp hit against the negative control dataset were classified as true positives and those that had no hit against the T3SEs dataset were classified as false positives. The sequences with hits in both datasets were further classified as follows. If a sequence had three or more hits in both datasets, a t-test was performed to evaluate the significance of sequence similarity to the T3SEs dataset in comparison with the negative control dataset (*p* < 0.05). If the number of hits was less than three in either of the datasets, the bitscore of BLASTp was used. If the lowest bitscore against theT3SE alignment was at least 1.5-fold larger than the maximum bitscore against the negative-control alignment, the sequence was considered true positive, and otherwise, false positive. The parameters of this pipeline were determined so that known effectors in T3SS1 and T3SS2 clusters were accurately classified as true positives.

### 2.4. Genomic Island Prediction and Gene Order Analysis

A dataset of 12 different *Vibrio* T3SS clusters (4 each from T3SS1, T3SS2, and T3SS3) was used. Genomic islands in the *Vibrio* genomes were predicted using IslandViewer 4 [27] and their T3SS gene clusters were assigned to their corresponding positions within the genomes. Then, T3SS gene clusters in all genomes were reannotated using Prokka with default parameters [28] and the annotations were manually revised to fit the unified nomenclature of T3SS proteins and some of them were abbreviated to fit in the figures. In addition, transposase, integrase, and phage-related genes were manually searched within 50-kb regions. The order and orthology of genes in each T3SS gene cluster was visualized using Clinker [29].

## 3. Results

### 3.1. Identification of T3SS3 Clusters from Protein Similarity

We first searched the set of 10 conserved proteins (SctC, D, J, N, Q, R, S, T, U, V) against the publicly available genomes, and identified 208 bacterial strains that possess all of them (Supplementary Table S1). Among the 10 proteins, SctD was excluded from further analyses due to the lack of its complete sequence in many bacterial strains. T3SS clusters that were confirmed to be located on a plasmid were also excluded to keep the phylogenetic analysis evolutionarily clearer. T3SS-possessing strains including *Yersinia* spp., *D. vulgaris*, *Grimontia hollisae* and *Vibrio mimicus* were excluded from our analysis at this stage.

From the remaining strain set, only one strain was kept for each species to eliminate duplicate information and to reduce the volume of phylogeny. Thus, we obtained a dataset of 9 core proteins from 12 *Vibrio* T3SS clusters (two sets from *V. pectenicida* 99-46-Y, *V. aquimaris* strain THAF100, *V. coralliilyticus* OCN008, two sets from *V. parahaemolyticus* RIMD 2210633, *V. cholerae* strain SA3G, *V. tubiashii* ATCC19109, *V. harveyi* E385, *V. alginolyticus* NBRC 15630, undefined *Vibrio* spp. THAF191c and 2521-89) and the other bacteria (Supplementary Table S2). The amino acid sequence conservation rates of the four core proteins (SctN, SctS, SctU and SctV) were > 35% in this dataset, whereas the conservation rate of three proteins (SctC, SctJ and SctQ) were lower.

**Figure 2:**
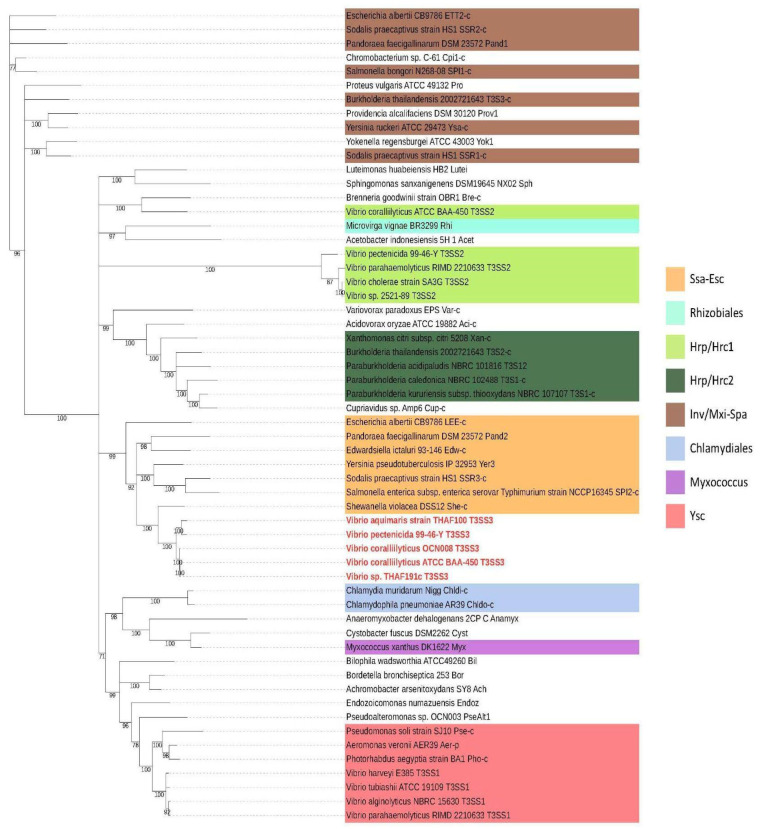
Phylogenetic tree of concatenated SctN-SctS-SctU-SctV proteins for bacteria with T3SS. *Vibrio* strains possessing a T3SS3 cluster are marked in red. *V. coralliilyticus* ATCC BAA-450 was added, in addition to the well-studied OCN008 strain, as the type strain possessing T3SS. Background colors show 8 T3SS families previously proposed for a subset of species analyzed [6,8].

To create a reliable phylogenetic tree that represents the evolutionary picture of T3SS clusters, concatenated sequences of highly conserved proteins (SctN, SctS, SctU and SctV of >35% identity) were used to classify T3SS families for the 48 bacterial species. The resulting tree was confirmed to be compatible with previous studies (Figure 2) [6,8].

In the phylogenetic tree, upstream branches were not stable, but the Vib-T3SS1 and Vib-T3SS2 were found within the Ysc and Hrp/Hrc1 families, respectively, as previously reported. In addition, sequences from 4 *Vibrio* strains (*V. pectenicida* 99-46-Y, *V. aquimaris* strain THAF100, *V. coralliilyticus* OCN008, unidentified strain THAF191c) were located within the Ssa-Esc family. These four strains were all isolated from seashells or corals and not from human patients. This new T3SS family is called T3SS3 in this report. To explicitly specify the source organism of these clusters, we also use the notation Vib-T3SS1/2/3.

### 3.2. Genomic Location and Gene Order of T3SS3 Gene Clusters

**Figure 3:**
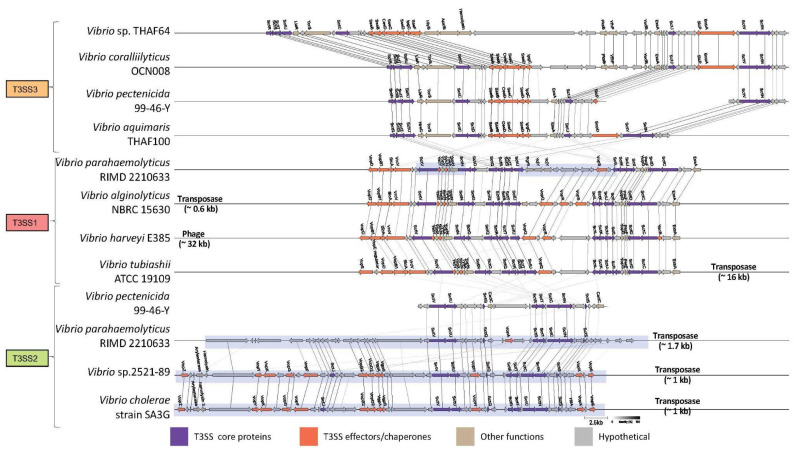
Gene orders of the T3SS1/2/3 clusters. Arrow direction and color correspond to respective coding strand and gene function, respectively. Arrow size reflects amino acid length, and the lines between arrows represent orthologs as computed by the Clinker software (see Methods). Genomic island is visualized as a blue shade. Genomic positions are shown in Supplementary Table S3.

Within *Vibrio* genomes, T3SS1 and T3SS3 clusters were located outside of genomic islands on the larger chromosome (chromosome I) according to the prediction by IslandViewer 4 (see Methods). In contrast, the T3SS2 clusters were mostly located within a genomic island, probably on chromosome II. For example, *V. parahaemolyticus* possesses its T3SS1 cluster on chromosome I, and its T3SS2 cluster in a genomic island on chromosome II (Figure 3). A possible exception was *V. pectenicida* 99-46-Y, whose T3SS2 cluster was not associated with transposase and was not located within the genomic island, although it should be noted that the *V. pectenicida* genome was not closed.

Within each of the T3SS1/2/3 clusters, the injectisome genes and secretory genes exhibited well conserved ordering, with only a handful of insertions or deletions (Figure 3). In particular, the genes encoding SctC-SctD (basal body) and SctR-SctS-SctT-SctU (export apparatus) were found in exactly the same consecutive order in all the investigated T3SS1 and T3SS3 sequences, but not in the T3SS2 sequences. The gene order of T3SS2 clusters was shuffled entirely when compared with that of T3SS1 or T3SS3 clusters. Three out of four T3SS2 sequences were computationally predicted to be on genomic islands, but the gene order and composition were very well conserved within the T3SS2 group.

In contrast to the different ordering and less sequence similarity of core injectisome proteins between T3SS1 and T3SS2, they shared a similar repertoire for effector proteins. Vop (*Vibrio* outer membrane) proteins dominate among the predicted gene functions in T3SS1 and T3SS2 clusters. On the other hand, effectors in T3SS3 were mostly similar to Sse proteins for *Salmonella* secretory effectors and no Vop annotation was assigned in the neighborhood of injectisome core proteins.

**Figure 4:**
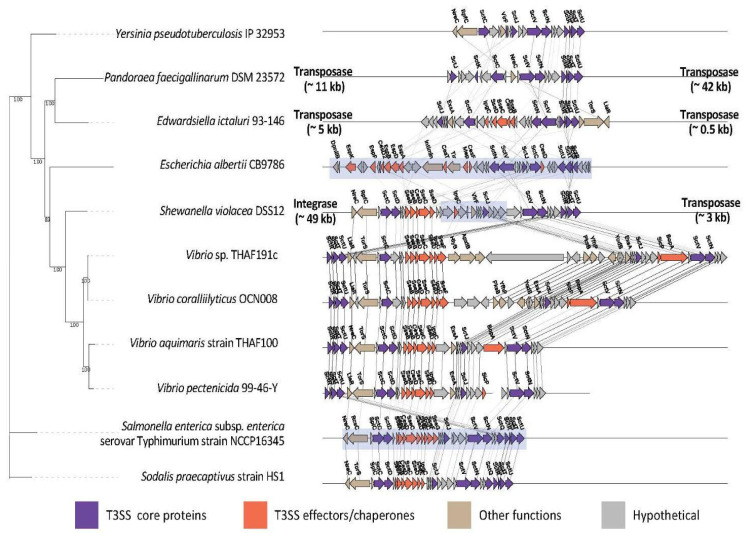
Phylogenetic tree of concatenated SctN-SctS-SctU-SctV proteins and the gene order of the Ssa-Esc family. Graphical notation is the same as Figure 3.

The gene order in Vib-T3SS3 was roughly conserved in several other bacteria of different orders in the Ssa-Esc family: *Shewanella violacea* DSS12 in Shewanellaceae (Alteromonadales)*, Salmonella enterica* subsp. *enterica* serovar Typhimurium strain NCCP16345 in Enterobacteriaceae (Enterobacterales), and *Sodalis praecaptivus* strain HS1 in Pectobacteriaceae (Enterobacterales) (Figure 4). In these strains, the conservation of gene order spanned not only for core injectisome proteins (SctC-SctD and SctR-SctS-SctT-SctU), but also for translocons (SseB-SseC-SseD) and chaperones (CesD-IpgC). However, the clusters of *Escherichia albertii* CB9786, *Edwardsiella icutaluri* 93-146 in Enterobacteriaceae, and *Pandoraea faecigallinarum* DSM 23572 in Burkholderiaceae exhibited different gene ordering. They also did not share *Salmonella* effectors or translocons.

Regarding genomic location, the T3SS genes in *S. violacea* DSS12, *S.* Typhimurium strain NCCP16345 and *E. albertii* CB9786 were found on a genomic island. In *S. violacea* DSS12, the cluster was sided by integrase and transposase genes. For the investigated Vib-T3SS3 clusters, no transposase/integrate genes were found in their 50 kb neighborhood.

### 3.3. Prediction of Putative T3SS3 Effectors, Translocons, and Chaperones

The gene order analysis focused on the neighborhood of injectisome genes, but effectors are often encoded outside of the cluster. In order to confirm the similarity of T3SS effectors (T3SEs) between Vib-T3SS3 and those in *Salmonella* spp., the sequences of experimentally validated effectors, translocons and chaperones were collected from previous studies [7,15,25] (see Methods). A dataset of 205 experimentally validated T3SEs and related proteins (Supplementary Table S4) was obtained.

Two hundred and five proteins were searched against the HMMER profiles of 3673 homologous gene groups created from protein sequences in the four *Vibrio* genomes with T3SS3 clusters, and 35 groups showed significant hits (E-value: <1 ×10^−3^). In order to minimize the number of false positives, the protein sequences of *Aliivibrio fischeri* and *Escherichia coli* K-12 were used as the negative control (see Methods). The combination of BLASTp searches of the 35 homologous groups against both 205 positive- and the negative-control datasets identified 14 true-positive protein groups (Table 2 and Supplementary Table S5). Eleven out of the 14 groups were similar to effectors, translocons, or chaperones in the Ssa-Esc family, just like the core T3SS proteins of T3SS3. The remaining few sequences were common with proteins in the Ysc or Inv/Mxi-Spa families. *Salmonella* translocons (SseB, SseC, SseD), EPEC/EHEC chaperone (CesD), and some core effectors in *Salmonella* (SseF, PipB) were conserved across Vib-T3SS3 strains. Not all the core *Salmonella* effectors, however, were detected in Vib-T3SS3 clusters in our analysis. For example, Jennings et al. defined seven core *Salmonella* effectors shared by all serovars (SseF, SseG, PipB, SteA, SteD and PipB2) [3], but the majority were completely absent from these *Vibrio* species (Table 2).

## 4. Discussion

### 4.1. Evolutionary Distance of T3SS3 from T3SS1 and T3SS2

Our bioinformatics analysis described the third variation of T3SS in *Vibrio*, here called T3SS3. From the sequence similarity of injectisome proteins, the new variant belongs to the Ssa-Esc family, and its T3SS effectors are also more similar to those in *Salmonella* spp. than those in previously identified T3SS1 and T3SS2 effectors (Table 2). Additionally, from the gene ordering, T3SS3 resembled the Ssa-Esc family. Between *Vibrio* species, T3SS3 and T3SS1 are more closely related in terms of sequence similarity and gene order in comparison with T3SS2, which resides in genomic islands (Figure 3). The T3SS2 cluster in *V. pectenicida* 99-46-Y was not predicted within a genomic island in our analysis, but this is probably a consequence of incomplete genome information.

Within the Ssa-Esc family, the T3SS3 clusters showed a gene composition and ordering similar to intestinal pathogens such as *Salmonella* and *Sodalis* (Figure 4, Table 2). In addition to SPI-2 T3SS in *Salmonella*, one representative T3SS cluster in the Ssa-Esc family is that of enteropathogenic *E. albertii* CB9786 [38]. This cluster resides within a LEE pathogenicity island in the chromosome and contributes to its pathogenicity. Its T3SS gene order is very different from those of Vib-T3SS3 clusters as well as *Salmonella*, *Sodalis*, and *Shewanella* strains in the Ssa-Esc family. This cannot be easily explained as the result of shuffling in the genomic island, because T3SS clusters in *Salmonella* and *Shewanella* are also within genomic islands. This highlights an interesting similarity between these strains and T3SS3-possessing *Vibrio* strains across different bacterial phyla.

Our analysis relies on the phylogenetic tree created from the four most conserved proteins (SctN, SctS, SctU and SctV) of the injectisome across bacteria with T3SS. The SctN is the ATPase to translocate effector proteins through the narrow T3SS syringe and is the most conserved component of the entire system (Figure 1). The remaining three proteins compose the export apparatus and their amino acid conservation rate across strains was >35%. The phylogenetic analysis supported the previously proposed classification of T3SS families, with Vib-T3SS1 and Vib-T3SS2 clusters being clustered within the Ysc and Hrp/Hrc1 families, respectively (Figure 2). Hu et al. [16] presented a different classification based on the phylogeny of a set of core T3SS proteins and their conserved syntenic orders. The resulting 13 classes were not compatible with the traditional T3SS families, but also exhibited a unique position of *Vibrio coralliilyticus* RE98 within the *Escherichia* LEE and *Salmonella* SPI-2 group, corresponding to our T3SS3. We created our phylogeny to keep its consistency with known T3SS families for easier functional studies. Our tree shape kept a similar classification, even if we added a few more conserved proteins (e.g., SctR), but the clean clustering was lost when we used all 10 conserved proteins.

### 4.2. Effect of Mobile Elements and Horizontal Gene Transfer

Gene order and location are important indicators of genetic exchange in bacteria. Because the computational prediction of genomic islands might not always be accurate, we manually searched mobile element-related genes within ~50-kb regions around each T3SS gene cluster in this study. Within this range, all T3SS3 clusters were free from transposes or integrases, whereas the cluster in *S. violacea* DSS12 was associated with mobile genes. The cluster in *S.* Typhimurium strain NCCP16345 was also mobile gene-free, but the entire cluster was known to be within the SPI2 pathogenicity island. Although our detailed analysis surveyed only a handful of genomes, this suggests that the T3SS3 clusters may be horizontally transferred between *Vibrio* and other bacterial species such as *Salmonella* and *Shewanella* sharing a common habitat. Indeed, the food poisoning pathogen *S.* Typhimurium is occasionally found in molluscan shellfish such as mussels and clams [39]. Similarly, *Shewanella* is found in marine environments as a symbiont of squid and corals [40]. *Vibrio* species also live in marine and freshwater environments, and *Vibrio* strains with T3SS3 clusters have been isolated from mollusks and corals. The direction of the horizontal gene transfer, however, is difficult to clarify only from the current bacterial genomes.

### 4.3. Repertoire of Effectors, Translocons, and Chaperones in T3SS3

To gain more understanding of the infection mechanism of strains with T3SS3, we selected candidate T3SS3 effectors in a rather strict way (Table 2). Most of the predicted T3SS3 proteins were homologous to those in *Salmonella*. Of note were the core effectors shared by multiple strains with T3SS3, e.g., the orthologs of SseF and PipB proteins in *Salmonella*. These effectors are associated with *Salmonella*-containing vacuoles (SCVs) and *Salmonella*-induced filaments (SIFs); they contribute to the intracellular life of *Salmonella* in host cells. This was unexpected because well-known *Vibrio* species such as *V. parahaemolyticus* and *V. cholerae* are historically considered extracellular pathogens [7,41]. There has been no report for species with T3SS3 that suggests they internalize into host cells.

The list of candidate effectors alone, however, is insufficient to suggest the infection pathway of the strains with T3SS3. The selected 15 putative protein groups (effectors, translocons and chaperones) are not the full set but rather a partial list. To clarify the true function of each protein, experimental evidence is essential. The number and type of effectors are known to differ even among the same species [3], and a recent report also indicates diversity of effectors in T3SS2 clusters [42]. Bioinformatics analysis can find many putative effectors as in our study, and their functional characterization through experiments is awaited [43].

In summary, we described a new variation of the T3SS gene cluster in *Vibrio* species. This group, T3SS3, shares core and related proteins with *Salmonella* and other enteropathogenic species that infect a wide range of marine and terrestrial hosts. Of note is the similarity of translocons, chaperones, and effectors to those in *Salmonella,* although the mode of infection is considered very different between *Vibrio* and *Salmonella*. Further experimental analyses on its effectors will provide more information about their functions and mechanisms of action in comparison with their Ssa-Esc counterpart in other bacterial species.

## Figures and Tables

**Table 1:** T3SS family classification [6,8].

T3SS Family	Known Members	Known Relevant Hosts
Ysc	*Yersinia enterocolitica* *Yersinia pestis* *Yersinia pseudotuberculosis*	Humans, rodents, insects
	*Pseudomonas aeruginosa* *Pseudomonas mosselii* *Pseudomonas otitidis*	Humans, plants, insects
	*Aeromonas* spp.	Humans, fish
	***Vibrio alginolyticus*** ***Vibrio harveyi*** ***Vibrio parahaemolyticus***	Humans, fish, mollusks
Inv/Mxi-Spa	*Yersinia ruckeri*	Salmonid fish
	*Salmonella enterica* *Salmonella bongori*	Humans
	*Shigella boydii*	Humans
	*Burkholderia* spp.	Mammals
Ssa-Esc	*Salmonella enterica*	Humans
	*Yersinia pseudotuberculosis* *Yersinia enterocolitica* *Yersinia kristensenii*	Humans, rodents
	*Edwardsiella ictaluri*	Humans, fish
	***Vibrio aquimaris*** ***Vibrio coralliilyticus*** ***Vibrio pectenicida***	Fish, mollusks
Hrp/Hrc1	*Erwinia amylovora*	Plants
	*Pantoea agglomerans*	Plants
	*Pseudomonas syringae*	Plants
	***Vibrio cholerae*** ***Vibrio parahaemolyticus*** ***Vibrio pectenicida***	Humans, fish, mollusks
Hrp/Hrc2	*Ralstonia solanacearum*	Plants
	*Xanthomonas* spp.	Plants
	*Burkholderia* spp.	Plants
Chlamydiales	*Chlamydia muridarum* *Chlamydophila pneumoniae*	Mammals, birds, protists
Rhizobiales	*Rhizobium* spp.	Leguminous plants
Myxococcales	*Myxococcus* spp.	
Desulfovibrionales	*Desulfovibrio vulgaris*	Humans

**Table 2:** Putative T3SS3 effectors, translocons, and chaperones. The ‘Average identity’ column shows amino acid conservation rates in the Vib-T3SS3 strains. The letters in the ‘Strains’ column indicate the presence of a homolog (A: *V. aquimaris* strain THAF100; C: *V. coralliilyticus* OCN008; P: *V. pectenicida* 99-46-Y; and T: *Vibrio* sp. THAF191c).

Protein Names	T3SS Family	Average Identity %	Strains	Biochemical and Cellular Functions
SseF	Ssa-Esc	29.5	A, C, P	Core effector in all pathogenic *Salmonella* serovars, tethering *Salmonella*-containing vacuoles (SCVs) to the Golgi network. SseF functions with another core effector, SseG protein [3].
SspH2	Ssa-Esc	32.5	T	Effector (E3 ubiquitin ligase) in pathogenic *Salmonella,* interfering with host immune signaling. SspH2 is associated with SspH1 and SlrP, which also show E3 ligase activity [3].
SseJ	Ssa-Esc	33.4	A, P	Effector (acytransferase) in pathogenic *Salmonella,* preventing collapse of microtubules to provide a solid network around SCVs [3,30].
PipB	Ssa-Esc	32.4	A, C, P, T	Core effector of unknown function in all pathogenic *Salmonella* serovars, localizing to SCVs and SIFs. Its associated protein PipB2 controls the kinesin-1 motor protein of host cells [3].
SopD2	Ssa-Esc	33.3	A, C	Effector that prevents from directing SCVs into late endosomes and lysosomes [3,30]
SopD	Ssa-Esc Inv/Mxi-Spa	18.9	A	Effector that promotes plasma membrane scission and the generation of SCVs [30]
CesD	Ssa-Esc	39.5 25.4	A, C, P, T	T3SS chaperone in enteropathogenic *E. coli* strains for more efficient secretion [31,32]
CesT			C, T	
SseB	Ssa-Esc	37.7 30.3 28.4	A, C, P, T	Translocon proteins in *Salmonella* strains to transfer T3SS effectors [33]
SseC				
SseD				
BopA/IcsB	Inv/Mxi-Spa	23.4	C, T	Effector in *Shigella* or *Burkholderia* strains helping to evade the host autophagy defense system [34]
BopC	Inv/Mxi-Spa	44.5	A, P	Effector in *Bordetella* strains contributing to the necrotic cell death of mammalian host cells [35]
ExoY	Ysc	31.2	A, P	Common effector (adenylate cyclase) in clinically isolated *Pseudomonas aeruginosa*, delaying the inflammatory pathways in mammalian host cells [36]
Scc2	Chlamydiales	27.5	C	T3SS chaperone specific to *Chlamydia* species and is similar to SycD of *Yersinia*, SicA of *Salmonella*, and IpgC of *Shigella* [37]

## Data Availability

All genetic data are available from the GenBank/ENA/DDBJ repository. Accession numbers of all strains are summarized in Supplementary Table S1.

## Supplementary Materials

The following supporting information can be downloaded at: https://www.mdpi.com/article/10.3390/microorganisms11020290/s1. Supplementary Table S1: Genomes containing T3SS; Table S2: T3SS core proteins; Table S3: genomic positions of T3SS; Table S4: validated effectors; Table S5: putative T3SS3 effectors.
